# Adrenohepatic Adhesion/Fusion Mimicking Hepatic Invasion: Two Cases of Benign Right Adrenal Lesions and a Review of the Literature

**DOI:** 10.3390/diagnostics16142297

**Published:** 2026-07-22

**Authors:** Rastko Zivic, Milos Petrovic, Nemanja Pijanovic, Miljan Milanovic, Stefan Mitic, Vladan Zivaljevic

**Affiliations:** 1 Clinic for Surgery, Clinical Center “Dr Dragisa Misovic-Dedinje”, 11000 Belgrade, Serbia; 2 School of Medicine, University of Belgrade, 11000 Belgrade, Serbia; 3 Clinic for Internal Medicine, Clinical Center “Dr Dragisa Misovic-Dedinje”, 11000 Belgrade, Serbia; 4 Clinic for Endocrine Surgery, University Clinical Center of Serbia, 11000 Belgrade, Serbia

**Keywords:** adrenal surgery, adrenohepatic fusion, adrenohepatic adhesion

## Abstract

Background and Clinical Significance: Adrenohepatic fusion (AHF) represents a developmental anatomical variant chracterised by direct continuity between the right adrenal cortex and hepatic parenchyma, often accompanied by partial or complete absence of the intervening fibrous capsule and periadrenal fat plane. In contrast, adrenohepatic adhesion (AHA) denotes a close apposition of the two organs with a shared capsule, but without actual parenchymal intermingling. AHF/AHA becomes most clinically relevant when a lesion arises in the right adrenal gland. In this setting, cross-sectional imaging frequently demonstrates apparent continuity of the lesion across the interface. These features are conventionally interpreted as signs of local invasion, and this may lead to diagnostic misclassification as hepatocellular carcinoma, adrenocortical carcinoma, or metastatic disease, resulting in unnecessarily extensive hepatectomy in cases of benign disease. Case presentation: Herein we report two female patients, 27 and 63 years old, who underwent right antero-lateral laparoscopic adrenalectomy. In the first patient, a heterogeneous, partly calcified haemorrhagic cyst was connected to the hepatic capsule, and AHA was the final finding. In the second patient, a cortical adenoma at the liver–adrenal interface was diagnosed as AHF. Conclusions: AHF and AHA should be considered when right adrenal mass is inseparable from hepatic segment VI or VII, particularly when other imaging and biochemical characteristics do not support malignancy. Close coordination between radiology and pathology can help reduce unnecessary liver resections, improve surgical strategies, and support appropriately conservative adrenal resection.

## 1. Introduction

Adrenohepatic fusion (AHF) represents a developmental anatomical variant characterised by direct continuity between the right adrenal cortex and hepatic parenchyma, often accompanied by partial or complete absence of the intervening fibrous capsule and periadrenal fat plane [1,2]. In contrast, adrenohepatic adhesion (AHA) denotes a close apposition of the two organs with a shared capsule, but without actual parenchymal intermingling [3]. According to autopsy studies, the frequency of AHF occurrence ranges from 1 to 28.1%, with an average of 5.6% [1,2,4,5]. AHA is mentioned in surgical and autopsy studies with a frequency ranging from 14.3% to 31.1% [5,6]. More recent reports in the literature suggest that these anomalies may be under-recognised in clinical imaging, particularly in patients undergoing evaluation for adrenal incidentalomas or suspected hepatic lesions. Despite this histopathological distinction, both entities are frequently conflated in clinical practice due to overlapping radiological and intraoperative features [7].

In such cases, on standard imaging procedures, loss of the fat plane between these organs may raise the suspicion of primary carcinoma of the liver or adrenal gland [8,9,10]. This is of major practical importance as it can influence preoperative planning. Intraoperative findings of adrenohepatic fusion may leave the surgeon uncertain as to whether to extend the extent of the operation. This is especially important in minimally invasive surgery where the lack of a clear dissection plane can lead to hepatic injury and bleeding which can make a clear intraoperative assessment very difficult. As a result, in such situations a surgeon may decide to convert to open surgery or, even, to perform unnecessary hepatic resections in benign tumours [8,9].

Although direct endocrine or metabolic consequences of AHF have not been consistently demonstrated, the presence of such aberrant microanatomical interfaces may influence local tissue architecture and imaging appearance. The clinical relevance is therefore mainly diagnostic and surgical, rather than functional.

The specific aim of this report is to highlight the diagnostic pitfalls created by AHA and AHF, and to reduce unnecessary hepatectomy for benign adrenal disease. To this end, we describe two benign adrenal lesions associated with AHA and AHF, correlate their imaging, intraoperative and histological findings, and summarise the practical diagnostic considerations for radiologists, surgeons and pathologists. When discussing the preoperative findings, we use AHA/AHF because the final diagnosis is made by histological assessment.

### Method of Literature Review

We performed a narrative literature review to support the Discussion section. PubMed was searched for “adrenohepatic fusion”, “adreno-hepatic fusion”, “adrenohepatic adhesion” and “ectopic adrenal gland liver”. We also screened the reference lists of relevant review papers. Because the literature consists of individual case reports and small series, the literature assessment was not designed as a formal systematic review or a meta-analysis. This report was prepared in accordance with the CARE (CAse REport) reporting guideline, and a completed CARE checklist is provided in Supplementary Materials.

## 2. Case Presentation

We describe two women, aged 27 and 63 years, both with benign right adrenal lesions associated with adrenohepatic fusion/adhesion, in whom we performed minimally invasive right anterolateral transperitoneal adrenalectomy. A timeline of both cases is summarised in Table 1.

### 2.1. Patient 1

A 27-year-old woman was admitted with an incidental finding of a potentially tumorous growth in the right adrenal gland on computed tomography. She had occasional and moderate pain in the right flank, no comorbidities, no previous operations, no allergies, and a negative family history of endocrine diseases. Physical examination showed no characteristic features. The patient presented with a BMI of 21.63 kg/m^2^ and was classified as ASA II. Baseline ECG, laboratory tests and chest radiograph were within normal limits. Overnight dexamethasone suppression test, serum cortisol, ACTH, metanephrine and normetanephrine levels were normal, as were potassium levels and the renin–aldosterone ratio. The enhanced CT scan showed a round, clearly demarcated, expansive lesion measuring 51 × 49 × 48 mm originating from the right adrenal gland and intruding into the liver parenchyma. In the native CT scan, the lesion was heterogeneous, with marginal hyperdensity (up to 50 HU) and an area of a partial marginal calcification (Figure 1). Postcontrast imaging revealed discrete density enhancement in a small cranioposterior part of the lesion. From a radiological perspective, the lesion demonstrated features that were not entirely characteristic of a typical adrenal adenoma. The heterogeneous density and the presence of calcifications raised the possibility of a chronic organised process, while the apparent extension into hepatic parenchyma suggested a more aggressive lesion. Importantly, the absence of a clear fat plane between the adrenal gland and the liver was interpreted as a potential sign of invasion. The differential diagnosis at this stage included organised haematoma, atypical adrenal adenoma and—less likely—adrenal cortical carcinoma or metastatic disease. The inability to confidently exclude malignancy based on imaging alone played a decisive role in opting for surgical intervention.

Given the uncertain preoperative diagnosis, we opted for a minimally invasive surgical approach. A right antero-lateral transperitoneal laparoscopic adrenalectomy was conducted using a standard approach with the patient in the left lateral decubitus position. Four ports were distributed along the right costal arch: two 10 mm ports (camera and working port) and two 5 mm ports (liver retractor and working port). Intraoperatively, a hard, calcified tumour was found with tight adhesions to the surrounding fatty tissue and liver tissue and a clear anatomical plane towards the inferior vena cava. Adrenalectomy was performed with partial resection of the adherent hepatic capsule with no significant blood loss. Because the hepatic capsule was resected, a drainage tube was placed in the right adrenal lodge; this was removed on postoperative day two. The operation lasted 120 min. Postoperative recovery was uneventful. Six months after surgery, the patient remained asymptomatic, with no clinical or radiological signs of recurrence. Gross examination of the excised mass showed a cystic tumour measuring 65 × 60 × 30 mm, which was filled with haemorrhagic content (Figure 2). The cystic wall was calcified, measuring 3 mm in thickness. Microscopic examination showed liver tissue with hepatocytes of regular morphology arranged in beams, while the lower part showed typical tissue of the cortex of the adrenal gland (zona glomerulosa), with clearly delimited cells grouped into fine clusters of glomerular pattern (Figure 3). Between these two tissues there was a fused capsule formed by hypocellular connective tissue. AHA was the definitive pathohistological diagnosis.

### 2.2. Patient 2

A 63-year-old woman was admitted with an incidental finding in the right adrenal gland on computed tomography. She had no symptoms, no comorbidities, no previous operations, no allergies, and a negative family history of endocrine diseases. Physical examination showed no characteristic features. The patient’s BMI was 21.26 kg/m^2^, corresponding to ASA physical status II. ECG, routine laboratory panels and chest X-ray demonstrated no significant abnormalities. Overnight dexamethasone suppression test, serum cortisol, ACTH, metanephrine and normetanephrine levels were normal, as were potassium levels and the renin–aldosterone ratio. The MR finding indicated a nodular, clearly delimited structure at the level of the right adrenal gland which showed a drop in signal on the out-of-phase sequence and did not show diffusion restriction. The structure was hyperintense on both T2 HASTE and T2 HASTE FS sequences and according to its MR characteristics primarily corresponded to an adenoma of the adrenal gland, with a diameter of 38 mm, indenting the liver and the inferior vena cava without clear infiltration of the vein (Figure 4).

CT showed a clearly delimited, nodular change in the right adrenal gland measuring approximately 21 × 26 × 46 mm (AP × LL × CC), with low densities in native scan (mean value approx. 2 HU). This structure was in close contact with segment VI of the liver, without definite CT signs of infiltration (Figure 5).

The discrepancy between benign imaging features and suspicious anatomical positioning created a diagnostic dilemma. Although the absence of diffusion restriction and the presence of low attenuation values on CT supported a diagnosis of a benign process, the inability to definitively exclude malignancy led to a more extensive surgical approach.

The patient subsequently underwent laparoscopic right adrenalectomy with resection of the liver parenchyma in which the tumour was embedded. Intraoperatively, the lesion was found to be indenting the inferior vena cava and closely adhering to the liver, with no clear dissection plane, further reinforcing the preoperative suspicion of invasion. Considering the close relation to the vena cava, the tumour was first completely mobilised from the lateral side in order to achieve a clear dissection plane between the vein and the tumour, and to visualise the short adrenal vein which was clipped and cut. Consequently, the decision was made to extend the resection margin to include the adjacent hepatic tissue on the upper pole of the tumour. Intraoperative blood loss was moderate, with no need for blood transfusion. A drainage tube was placed in the right adrenal lodge; this was removed on postoperative day three. The operation lasted 175 min. The postoperative course was uneventful. Six months after surgery, the patient remained asymptomatic, with no clinical or radiological signs of recurrence. Six months after the surgery, the patient was fully recovered. Histological examination showed a direct contact of cortical adrenal tissue, i.e., cells with light, vacuolated cytoplasm (zona fasciculata) and hepatocytes organised in a normal trabecular arrangement, without signs of desmoplasia, necrosis or inflammation. Partial to complete loss of the fibrous capsule was seen, with consequent direct connection of adrenal and hepatic tissue, which is indicative of adrenohepatic fusion—AHF (Figure 6).

## 3. Discussion

From a developmental standpoint, AHF and AHA are best understood as disorders of incomplete embryological separation. The adrenal cortex originates from mesodermal coelomic epithelium, whereas the liver develops from foregut endoderm [11,12]. During normal embryogenesis, these structures undergo spatial segregation and encapsulation [13]. Failure of mesenchymal differentiation at the interface may result in persistent tissue continuity. This concept, originally proposed in classical pathological descriptions, remains the most plausible mechanistic explanation for the formation of AHF. The absence of a distinct capsule and fat plane between the two organs supports the concept of AHF/AHA as a developmental anomaly, as opposed to an acquired pathology. Importantly, the biological significance of AHF extends beyond anatomical variation. In [1], intra-adrenal bile ductules were described within fusion zones, suggesting that AHF represents true microanatomical admixture, rather than simple adhesion. This observation is especially relevant for pathology-oriented case reports, because it implies that AHF can create an aberrant tissue microenvironment in which hepatic and adrenocortical elements coexist across a fused boundary.

Clinically, AHF/AHA becomes most relevant when a lesion arises in the right adrenal gland. In such cases, cross-sectional imaging frequently demonstrates absence of a fat plane between the adrenal gland and liver, with apparent continuity of the lesion across the interface. These features are conventionally interpreted as signs of local invasion. However, in the context of AHF, they represent a pseudo-invasive phenomenon rather than true malignant infiltration.

Radiologically, lesions arising in AHF can exhibit heterogeneous enhancement patterns, irregular borders, and apparent intrahepatic localisation. These features may mimic hepatocellular carcinoma, adrenal cortical carcinoma or metastatic disease. It was previously demonstrated that the lack of a peri-adrenal capsule and intermingling of tissues produce a spectrum of imaging appearances, often leading to diagnostic uncertainty and misclassification [14].

Recent case series further emphasise the diagnostic challenge. A recent review discussed AHF-associated adrenal tumours and reported frequent instances of misdiagnosis, including cases initially interpreted as malignant due to apparent hepatic invasion. Specifically, a total of 16 cases with AHF from 14 published papers [15] were analysed. In six out of 16 cases, partial hepatectomy was performed along with adrenalectomy for benign adrenal diseases (cortical adenoma in five cases and adrenal tuberculosis in one case), which clearly indicates that the radical surgery that was conducted was unnecessary [8,9,16,17,18,19]. In three of these six cases, a tumour biopsy was performed preoperatively, raising the suspicion of hepatocellular carcinoma of the liver, which then justified the decision to perform a hepatectomy [9,17,18].

The surgical implications of this phenomenon are clinically important. During minimally invasive adrenalectomy, the absence of a clear dissection plane between the adrenal gland and liver may result in capsular injury, bleeding, or conversion to open surgery. Conversely, failure to recognise AHF may lead to incomplete adrenal resection due to intrahepatic extension of adrenal tissue. In a series of laparoscopic right adrenalectomies, adrenohepatic attachment contributed to incomplete adrenal resection in 11 of 27 procedures (40.7%), while histological intermingling of parenchymal cells was confirmed in 28.1% of the accompanying specimens [5].

Radiologists may reasonably suspect primary adrenal carcinoma invading the liver, hepatocellular carcinoma invading the adrenal gland, or a malignant hepatic mass of distant origin in cases where the right adrenal lesion is partially intrahepatic, subcapsular or inseparable from the inferior surface of the liver. However, a number of tissue formations associated with AHF have ultimately been shown to be benign (adrenocortical adenomas). Apparent invasion has frequently been found to be an imaging manifestation of pre-existing adrenal–hepatic continuity, rather than a sign of actual tumour aggressiveness [9,14,15,17,18].

AHF/AHA can also affect the diagnostic conclusion about tumour localisation and the apparent organ of origin. Reports of intrahepatic adrenocortical adenoma arising from AHF demonstrate that benign adrenal cortical neoplasms may present as hepatic masses. It has been suggested that performing a retrospective CT review could reveal continuity with the right adrenal limb, as well as low attenuation consistent with adrenal cortical tissue, while a biopsy should be considered when AHF-related intrahepatic adrenocortical adenoma is suspected, precisely to avoid unnecessary hepatic resection [8,18].

In practical terms, the most useful preoperative sign is not just the absence of a fat layer (which has multiple causes), but a clear connection to the right adrenal gland, combined with non-aggressive imaging features. Existing research indicates that lesions in AHF might still show signs that align with an adrenocortical origin, such as low attenuation when lipid-rich, proximity to the right adrenal gland, and a lack of the destructive features that are typical of advanced malignancies. Additionally, some imaging traits may help predict the presence of adrenohepatic adhesion before laparoscopic surgery, underscoring the importance of thorough preoperative imaging assessments [6].

Table 2 summarises the distinctive features of AHA and AHF.

Advanced imaging may reduce, but not eliminate, the diagnostic uncertainty. Chemical-shift MRI and adrenal-protocol CT scanning remain the most practical tools for identifying adrenal adenomas [19,20,21]. Hepatobiliary MRI with gadoxetate can help determine the presence of functioning hepatocytes within a lesion; however, adrenal PET tracers remain a highly specialised tool, limited by availability and spatial resolution [22,23,24]. None of these tools can reliably be used to ascertain the microscopic intermingling which is used to distinguish between AHA and AHF. For this, pathohistology is still required.

Our experience reiterates the notion that benign right adrenal lesions in AHF/AHA can be mistaken for liver invasion, leading to surgical challenges that may have serious consequences. Furthermore, the cases we discuss provide pathohistological insights supporting the pseudo-invasive concept. Because one lesion we dealt with was cystic/haemorrhagic and the other appeared benign but nonetheless raised radiological concerns, we thus expand the morphological understanding of benign adrenal pathology that may be complicated by AHF/AHA.

In summary, we recommend that AHF/AHA should be a part of the differential diagnosis whenever a right adrenal lesion seems to invade liver segments VI or VII without clear signs of extensive malignancy. Radiologists and surgeons should actively look for residual connections to the adrenal gland, scrutinise the imaging features for signs of adrenocortical origin, and interpret any apparent capsular breaches carefully. In uncertain cases, tissue sampling could be valuable, though it is vital to weigh the risks and limitations of biopsy. The central point is not that biopsies are always necessary, but that being aware of AHF shifts the considerations around assuming malignancy or legitimate liver invasion.

### 3.1. Patient Perspective

For this retrospective case report, formal patient perspective statements were not collected, and no standardised patient-reported outcome instrument was used. Both patients were reviewed in the outpatient clinic during the six-month follow-up period. Neither reported persistent pain, wound problems or limitation of daily activities, and both had returned to their usual daily activities after surgery. The absence of a structured patient-reported outcome assessment is acknowledged below as a limitation.

### 3.2. Strengths and Limitations

The strength of this report lies in its direct correlation of imaging, intraoperative assessment and pathohistology in two benign right adrenal lesions, ultimately leading to diagnoses of AHA and AHF. The report therefore illustrates the clinically relevant overlap between these two entities and provides practical assistance in reaching a diagnosis.

Our report has several limitations. It is based on only two patients from a single centre, so the findings are descriptive and cannot support conclusions about prevalence, outcomes or optimal management. No advanced molecular or functional imaging or germline/tumour genetic testing was performed, follow-up was limited to six months, and formal patient-reported outcome measures were not collected. The literature review which we conducted was narrative rather than systematic. Due to the retrospective nature of this report, and the small sample size involved, our findings should be interpreted with caution, and larger series are needed.

On a general level, the limitation of the current literature is that it largely relies on isolated case studies and small retrospective groups, making it hard to draw broad conclusions. Terminology can be inconsistent, and descriptions of pathological interfaces can lack detail or vary significantly. This complicates the determination of whether AHF and AHA are separate developmental entities or more mixed categories where surgical and pathological results do not always align. Still, the core patterns that emerge across available reports are quite consistent.

## 4. Conclusions

AHF/AHA represents a developmental abnormality at the adrenal–hepatic interface, where tissue continuity, a shared capsule and, sometimes, microscopic mixing create a pseudo-invasive appearance both in imaging studies and surgical findings. When benign right adrenal tumours arise in this context, they may be mistaken for malignant liver lesions or invasive tumours. Awareness of this interface phenomenon, together with the multidisciplinary correlation of imaging, operative and pathological findings, can prevent unnecessary hepatic resection while still achieving complete adrenal removal.

## Figures and Tables

**Figure 1:**
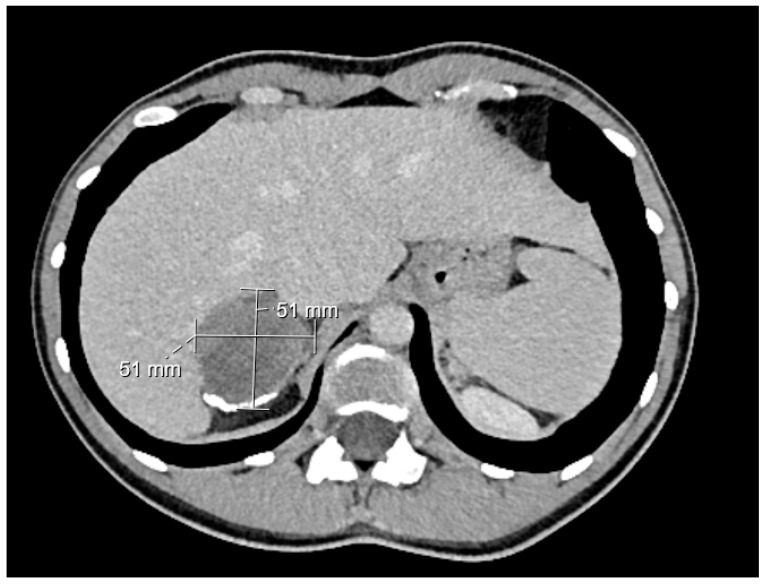
Abdominal CT of patient 1 (axial plane). The right adrenal lesion is heterogeneous, with partial marginal calcification and apparent extension towards the liver parenchyma, with loss of the intervening fat plane.

**Figure 2:**
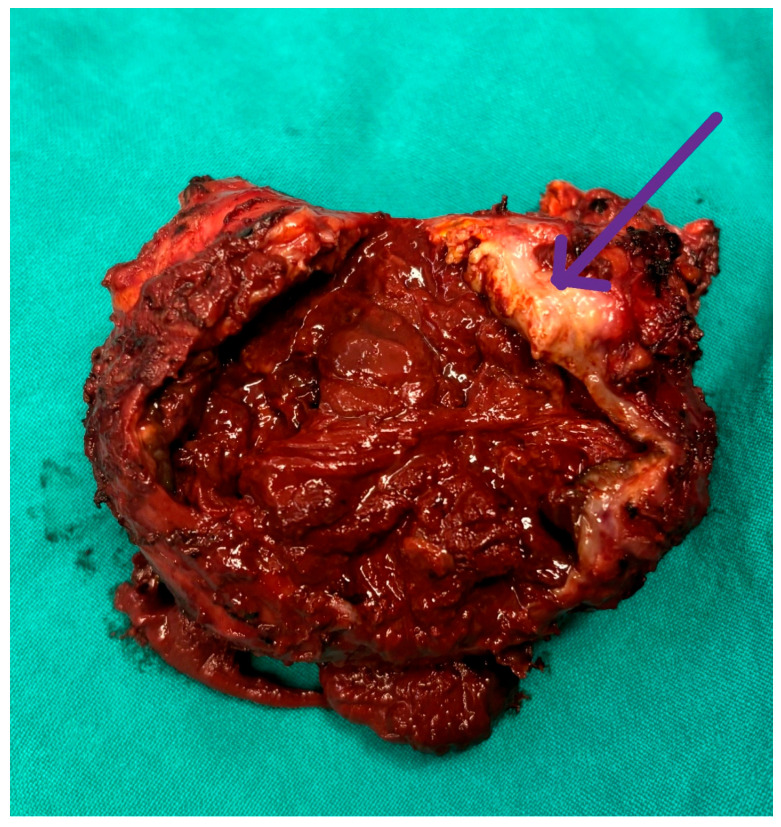
Gross appearance of the bisected right adrenal gland, revealing a haemorrhagic cyst with a calcified wall.

**Figure 3:**
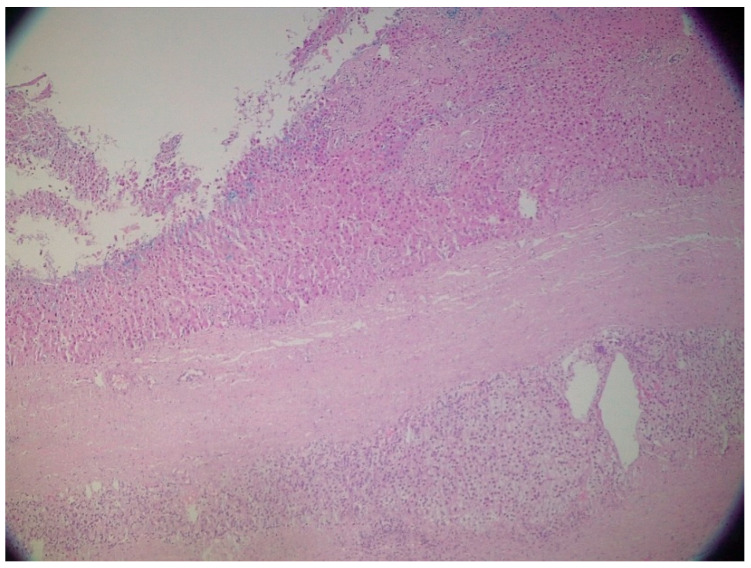
Light microphotograph of AHA (H&E, original magnification ×50). Hepatic parenchyma and adrenal cortex (zona glomerulosa) are separated by a fused capsule of hypocellular connective tissue, without parenchymal intermingling.

**Figure 4:**
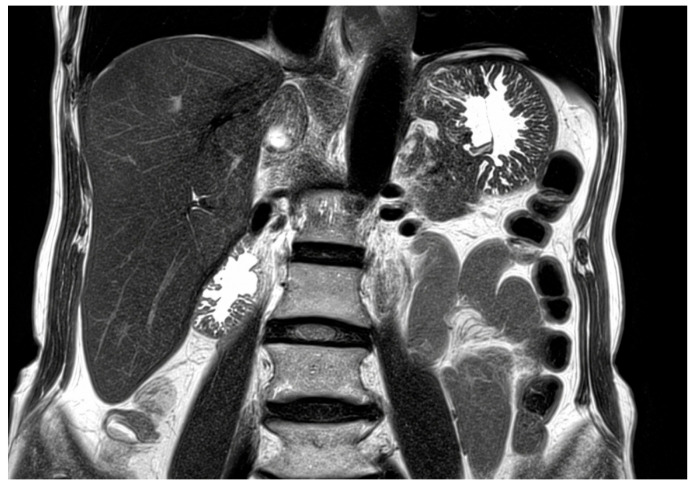
Abdominal MRI of patient 2 (coronal plane). A 38 mm right adrenal lesion with features of adenoma indents the liver and the inferior vena cava, without clear infiltration.

**Figure 5:**
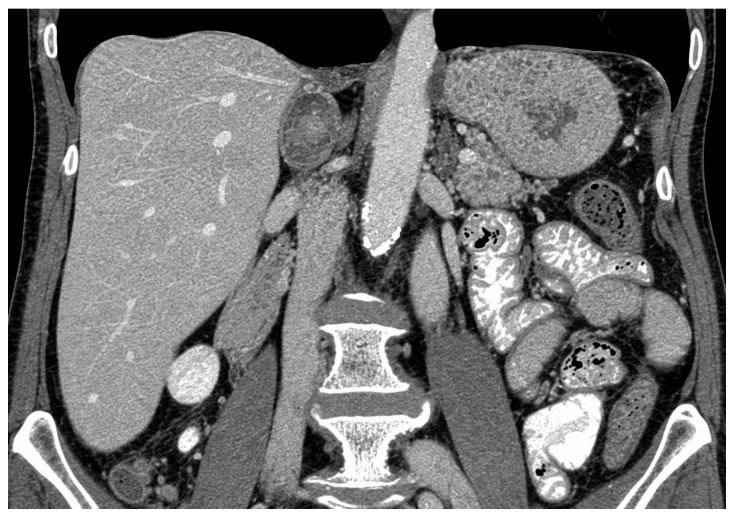
Abdominal CT of patient 2 (coronal plane). The adrenal lesion is in close contact with hepatic segment VI, without definite signs of infiltration.

**Figure 6:**
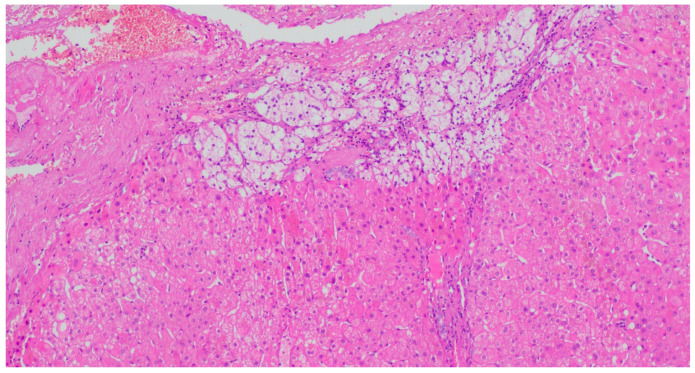
Light microphotograph of AHF (H&E, original magnification ×100), showing partial to complete loss of the fibrous capsule with direct continuity between hepatic and adrenal tissue.

**Table 1:** Timeline of the two cases.

Time Point	Patient 1 (27-Year-Old Woman)	Patient 2 (63-Year-Old Woman)
Presentation	Incidental right adrenal lesion on CT; occasional moderate right flank pain	Incidental right adrenal lesion on CT; asymptomatic
Endocrine work-up	Non-functioning: overnight dexamethasone suppression test, cortisol, ACTH, metanephrines, potassium and renin–aldosterone ratio all normal	Non-functioning: same panel, all normal
Imaging	CT: 51 × 49 × 48 mm heterogeneous, partly calcified lesion; loss of fat plane, with apparent extension into the liver	MRI: 38 mm lesion with features of adenoma; CT: 21 × 26 × 46 mm, close contact with segment VI, no definite infiltration
Preoperative differential diagnosis	Organised haematoma, atypical adenoma, adrenal cortical carcinoma or metastasis	Adrenal adenoma; malignancy not confidently excluded
Surgery	Laparoscopic right adrenalectomy with partial resection of the adherent hepatic capsule; 120 min	Laparoscopic right adrenalectomy with resection of the adjacent hepatic parenchyma; 175 min
Drain removal	Postoperative day 2	Postoperative day 3
Histopathology	Haemorrhagic cyst with a calcified wall; fused capsule without parenchymal intermingling; final diagnosis of AHA	Cortical adenoma; partial to complete capsular loss with direct adrenal-hepatic continuity; final diagnosis of AHF
Follow-up (6 months)	Asymptomatic; no clinical or radiological signs of recurrence	Asymptomatic; no clinical or radiological signs of recurrence

**Table 2:** Practical distinctions between adrenohepatic adhesion (AHA) and adrenohepatic fusion (AHF).

Adrenohepatic Adhesion (AHA)	Adrenohepatic Fusion (AHF)	Practical Implication
Close apposition or shared/fused (preserved) capsule No actual parenchymal intermingling	Partially or completely lost capsule Direct continuity or actual intermingling of adrenal cells and hepatocytes	Final distinction requires pathohistology Imaging and intraoperative findings may overlap
Loss of the fat plane Apparent capsular contact with the liver	Loss of the fat plane Adrenal tissue/tumour may appear intrahepatic or invasive	Both can mimic hepatic invasion/origin, especially in segments VI and VII.
Adheres densely to the hepatic capsule Difficult to dissect	True fusion zone No dissection plane	Risk of bleeding or unnecessary hepatic resection if this is not anticipated
Overinterpretation as malignant invasion	Overinterpretation as invasion or incomplete adrenalectomy	Preoperative multidisciplinary review is essential.

## Data Availability

The original contributions presented in this study are included in the article/Supplementary Material. Further inquiries can be directed to the corresponding author.

## Supplementary Materials

The following supporting information can be downloaded at: https://www.mdpi.com/article/10.3390/diagnostics16142297/s1, Table S1: CARE Checklist [25].
