# Supplementary material for: Adrenohepatic Adhesion/Fusion Mimicking Hepatic Invasion: Two Cases of Benign Right Adrenal Lesions and a Review of the Literature
Source: Diagnostics (Basel). 2026 Jul 22;16(14):2297. doi: 10.3390/diagnostics16142297 (PMC13409512; doi:10.3390/diagnostics16142297)
Supplement: Supplementary file 1 [file diagnostics-16-02297-s001.zip › diagnostics-4367197-supplementary.pdf]

Supplementary Material - CARE Checklist [1]

"Adrenohepatic Adhesion/Fusion Mimicking Hepatic Invasion: Two Cases of Benign Right Adrenal Lesions and Literature Review"

This report describes two patients. Where an item is reported separately for each patient, we provide both locations.

| No. | Topic | Checklist item | Reported | Location in manuscript |
|---|---|---|---|---|
| 1 | Title | The words "case report" (or "cases") appear in the title, together with the area of focus. | Yes | Title, p. 1 |
| 2 | Key words | 2 to 5 key words identifying the diagnoses or interventions in this case report. | Yes | Keywords, p. 1 (3 terms: adrenal surgery, adrenohepatic fusion, adrenohepatic adhesion) |
| 3a | Abstract | Introduction: what is unique about this case and what does it add to the literature? | Yes | Abstract, p. 1 |
| 3b | Abstract | Main symptoms and/or important clinical findings. | Yes | Abstract, p. 1 |
| 3c | Abstract | Main diagnoses, therapeutic interventions and outcomes. | Yes | Abstract, p. 1 |
| 3d | Abstract | Conclusion — the main take-away lesson(s). | Yes | Abstract, p. 1 |
| 4 | Introduction | Brief background summary of the case, referencing the relevant medical literature. | Yes | Introduction, pp. 1–2 |
| 5a | Patient information | De-identified demographic and other patient information. | Yes | Case presentation, pp. 3, 5; Table 1, p. 7 |
| 5b | Patient information | Main concerns and symptoms of the patient. | Yes | Patient 1, p. 3; Patient 2, p. 5; Table 1, p. 7 |
| 5c | Patient information | Medical, family and psychosocial history, including relevant genetic information. | Yes | Patient 1, p. 3; Patient 2, p.5 (no comorbidities, negative family history) |
| 5d | Patient information | Relevant past interventions and their outcomes. | Yes | Patient 1, p. 3; Patient 2, p. 5 (no previous operations) |
| 6 | Clinical findings | Describe the relevant physical examination and other clinical findings. | Yes | Patient 1, p. 3; Patient 2, p. 5 |
| 7 | Timeline | Relevant data from this episode of care organised as a timeline. | Yes | Table 1 "Timeline of the two cases", p. 7 |
| 8a | Diagnostic assessment | Diagnostic methods (physical examination, | Yes | Patient 1, p. 3; Patient 2, pp.5- 6; Table 1, p. 7 |

| | | | | |
|---|---|---|---|---|
| | | laboratory testing, imaging, questionnaires). | | |
| 8b | Diagnostic assessment | Diagnostic challenges. | Yes | Patient 1, p. 3; Patient 2, p. 5-6; Discussion, pp. 8-10 |
| 8c | Diagnostic assessment | Diagnostic reasoning, including differential diagnosis. | Yes | Patient 1, p. 3; Patient 2, p. 5-6; Table 1, p. 7; Table 2, p. 9 |
| 8d | Diagnostic assessment | Prognostic characteristics, where applicable. | Yes | Discussion, pp. 8–10 (benign nature; pseudo-invasion) |
| 9a | Therapeutic intervention | Types of intervention (pharmacologic, surgical, preventive, self-care). | Yes | Patient 1, p. 4; Patient 2, p.6; Table 1, p. 7 |
| 9b | Therapeutic intervention | Administration of the intervention (dosage, strength, duration). | Yes | Patient 1, p. 4; Patient 2, p.6 (operative technique, operative time) |
| 9c | Therapeutic intervention | Changes in the intervention, with explanations. | Yes | Patient 2, p. 6 (intraoperative decision to extend resection to adjacent hepatic tissue) |
| 10a | Follow-up and outcomes | Clinician- and patient-assessed outcomes, where appropriate. | Partly | Patient 1, p. 4; Patient 2, p. 6; Patients' perspective, p. 10. Clinician-assessed outcomes reported; no standardised patient-reported outcome instrument was used (stated as a limitation) |
| 10b | Follow-up and outcomes | Important follow-up diagnostic and other test results. | Yes | Patient 1, p. 4; Patient 2, p. 6; Table 1, p.7 (asymptomatic at 6 months, no signs of recurrence) |
| 10c | Follow-up and outcomes | Intervention adherence and tolerability, and how this was assessed. | Yes | Patients' perspective, p. 10 (outpatient review; no persistent pain, wound problems or limitation of activities) |
| 10d | Follow-up and outcomes | Adverse and unanticipated events. | Yes | Patient 1, p. 4; Patient 2, p.6 (postoperative course uneventful; none observed) |
| 11a | Discussion | Strengths and limitations of the management of this case. | Yes | Strengths and limitations, p. 10 |
| 11b | Discussion | Discussion of the relevant medical literature. | Yes | Discussion, pp. 8-10 |
| 11c | Discussion | The rationale for the conclusions. | Yes | Discussion, pp. 8–10; Conclusion, p. 10 |
| 11d | Discussion | The primary take-away lessons of this case report. | Yes | Conclusion, p. 10 |
| 12 | Patient perspective | The patient(s) should share their perspective on the care they received. | Partly | Patients' perspective, p. 10. Formal perspective statements were not collected for this retrospective report; this is stated transparently and acknowledged as a limitation |
| 13 | Informed consent | Did the patient give informed consent? Provide if requested. | Yes | Informed Consent Statement, p. 11 |

1. Gagnier, J.J.; Kienle, G.; Altman, D.G.; Moher, D.; Sox, H.; Riley, D.; the CARE Group. The CARE guidelines: consensus-based clinical case report guideline development. J. Clin. Epidemiol. 2014, 67, 46–51, https://doi.org/10.1016/j.jclinepi.2013.08.003.
